# The Role of Food and Nutrition System Approaches in Tackling Hidden Hunger

**DOI:** 10.3390/ijerph8020358

**Published:** 2011-01-31

**Authors:** Francesco Burchi, Jessica Fanzo, Emile Frison

**Affiliations:** 1 Department of Economics, Roma Tre University, Via Silvio D’Amico 77, 00145, Rome, Italy; E-Mail: fburchi@uniroma3.it (F.B.); 2 Bioversity International, Via dei Tre Denari 472/a, 00057 Maccarese, Roma, Italy; E-Mail: e.frison@cgiar.org (E.F.)

**Keywords:** food security, food and nutrition systems, hidden hunger, food-based strategies, undernutrition, agriculture

## Abstract

One of the World’s greatest challenges is to secure sufficient and healthy food for all, and to do so in an environmentally sustainable manner. This review explores the interrelationships of food, health, and environment, and their role in addressing chronic micronutrient deficiencies, also known as “hidden hunger”, affecting over two billion people worldwide. While the complexity and underlying determinants of undernutrition have been well-understood for decades, the scaling of food and nutrition system approaches that combine sustainable agriculture aimed at improved diet diversity and livelihoods have been limited in their development and implementation. However, an integrated system approach to reduce hidden hunger could potentially serve as a sustainable opportunity.

## 1. Introduction

At the Millennium Summit in September 2000, world leaders adopted the UN Millennium Declaration, committing their nations to a bold global partnership to reduce extreme poverty and to address a series of time-bound health and development targets [1]. Among these Millennium Development Goals (MDGs) is a commitment to reduce the proportion of people who suffer from hunger by half between 1990 and 2015 [2], as set in the first MDG, with two targets monitored globally.

Statistics show that hunger continues to be a dramatic problem in developing and emerging countries and that the progress towards the achievement of this goal is slow, with nearly 1 billion hungry people [3]. However, hunger and calorie deficits are only one part of the story. Many have access to the minimum amount of calories, but are deficient in one or more micronutrients. Micronutrient deficiencies, estimated to affect at least 2 billion people, are the cause of so-called “hidden hunger”. Since micronutrient deficiencies lead to a vast range of diseases and other health disorders, their decrease is likely to help the achievement of the other health-related MDGs such as Goals 4, 5 and 6 which call for reductions in child mortality, maternal mortality and prevalence of HIV-AIDS, malaria and other diseases, respectively.

This paper examines the concept of hidden hunger and tries to sort out possible strategies to tackle the problem. We review the recent history of agriculture and nutrition policies and programmes with a focus on recent food-based approaches to improving micronutrient deficiencies, and some insights into the possible benefits of a more “food and nutrition system” approach to combating hidden hunger.

## 2. A Needle in the Haystack: “Hidden Hunger”

There are nearly 925 million hungry people in the World [3]. This is a rough estimate based on the prevalence of people who are assumed to have an energy intake below that which is required to maintain body weight, body composition and levels of necessary and desirable physical activity for long-term good health. The number of those hungry is higher than that in 2007 as a partial consequence of the 2007–2008 food price crisis, and the overall recent economic crisis.

While the FAO is one of the most cited sources of data concerning hunger and food security, some experts disagree with the theoretical model in which these final numbers are obtained. The assumption is that hunger (often referred to as food security) is an exclusive outcome of the lack of sufficient calories for survival. The statistic is measured using a complex calculation which entails several steps: (1) identification of the total amount of food available in the country, through the “food balance sheet”, an input-output matrix where the quantities of food produced, traded, or eventually received under the form of food aid are registered; (2) subtraction of the quota of food which is assumed to perish and the one used for purposes different from human nutrition; (3) conversion of food into calories; (4) estimates of food distribution among households, first, and then within households based on household food, consumption or expenditure data; and (5) final estimates of the prevalence of hunger [4].

The FAO estimates show a decrease of those who are hungry during periods of increased national production because of assumed constant distribution of food nationally (household surveys are rarely undertaken, thus the distribution of calories is based on the previous survey). This emphasizes only the benefits of policies and programs focused on food availability, which we now understand is a limited view. Studies show that rising production may not have any impact on people’s *access* to food, and does not take into account the quality of food accessed or consumed [5,6].

**Figure 1.**
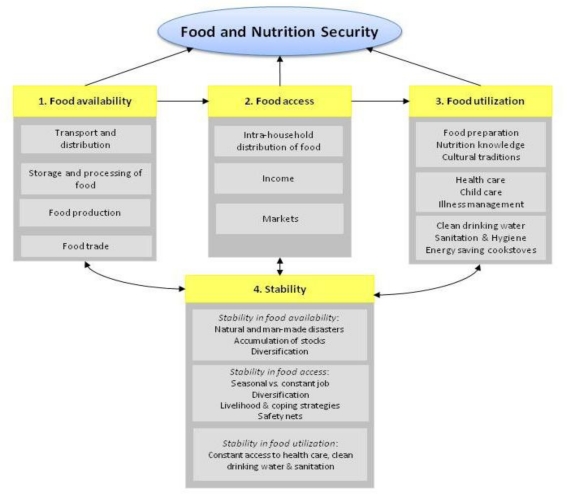
The four dimensions of food security.

The fact that hunger statistics deal only with caloric intake has been heavily criticized by nutritionists and by scholars advocating for a multi-dimensional idea of food security (Figure 1). A strictly calorie-based approach is incoherent with the widely accepted definition of food security given at the 1996 World Food Summit, according to which, “Food security exists when all people, at all times, have physical and economic access to sufficient, safe and nutritious food to meet their dietary needs and food preferences for an active and healthy life” [7]. The stress on “safe and nutritious food”, as well as the final goal to ensure “an active and healthy life” calls for a broader, though more complex, analysis of people’s diet.

What does it mean to be hungry? In its common usage, hunger describes the subjective feeling of discomfort that follows a period without eating [8]; however even temporary periods of hunger can be debilitating to longer term human growth and development [9]. We may further distinguish *acute* from *chronic* hunger: the first situation occurs when the insufficient intake of food is temporary, usually caused by an external shock, whereas the latter indicates a shortage of food on a persistent basis [10]. The term *undernourishment* defines insufficient food intake to continuously meet dietary energy requirements [11], with the FAO further defining hunger as the consumption of less than 1,600–2,000 calories per day. *Undernutrition* is commonly used to describe people who are malnourished if their diet does not provide adequate calories, protein for growth and maintenance, and micronutrients; or they are unable to fully utilize the food they eat due to illness. The concept of food security goes beyond caloric intake and addresses both hunger and undernutrition. Reducing levels of *hunger* places the emphasis on the quantity of food, and refers to ensuring a minimum caloric intake. Conversely, ensuring adequate *nutrition* refers to a diet’s quality. A diet rich in proteins, essential fatty acids, and micronutrients has been proven to improve birth weight, growth, and cognitive development while leading to lower levels of child mortality [12–16].

A lack of these essential vitamins and minerals often results in “hidden hunger” where the signs of undernutrition and hunger are less visible. A person may have access to a sufficient amount of calories but lack adequate micronutrients [17]. This phenomenon has been defined “hidden hunger” because its symptoms are not always obvious and people may not even be aware of it. Its negative, sometimes lifelong, consequences on health, productivity, and mental impairment, are devastating [18].

Micronutrients are nutrients (dominantly vitamins and minerals) required by humans throughout life in order to carry out a vast range of physiological functions. While it is argued that at least 51 different nutrients are needed in adequate amounts by human beings [19], there are 19 essential micronutrients “for physical and mental development, immune system functioning and various metabolic processes” [17]. Recent data highlight that an estimated 2 billion people suffer from one or more micronutrient deficiencies [19], demonstrating that hidden hunger is responsible in part for the global malnutrition burden. Most development programmes have focused on three micronutrients, which have devastating consequences for many: vitamin A, iron, and iodine.

The estimated numbers of individuals deficient in micronutrients are likely to underestimate the prevalence of the hidden hunger because of the actual presence of the various diseases associated with poor nutrition and the simplification of diets worldwide. Moreover, there are potentially many more people who do not have an adequate amount of other essential micronutrients such as vitamin B_12_, zinc and folate. Unfortunately, data concerning the prevalence of deficiencies in these micronutrients is limited. Zinc deficiency, for example, has proven difficult to quantify and statistics of global prevalence of zinc deficiency remain estimates [20].

## 3. History of Agricultural and Nutrition Policies

Traditionally, agriculture, nutrition and health sectors have operated as separate entities, and therefore policies and government structures have been designed without looking closely at the interactions among these sectors.

### 3.1. Agricultural Policies

Agricultural policies and projects have traditionally focused only on increasing yields, productivity, and general food availability in countries or regions, relevant in both developing and developed nations. As argued by Bouis and Welch, “Agricultural systems have never been explicitly designed to promote human health and, instead, mostly focus on increased profitability for farmers and agricultural industries” [21].

In an industrialized country such as the United States, national agricultural policies have rarely focused on public health [22,23]: since the 1930s their main aim has been to maintain or improve the living standards of farmers and their families, and to ensure availability of enough food to feed all Americans in the present and future times [24]. The standard federal food assistance programs have been largely focused on calorie-based approaches to improve nutrition with little attention to improving the quality of food. As the authors argue, “All of these programs appear to have been developed with the assumption that if consumers were provided with an adequate supply of food and calories, good nutrition would naturally result” [22].

In the context of developing countries, during the 1970s, the “Green Revolution” was launched in Asia and Latin America. Labor-saving technologies, fertilizers, pesticides and various types of improved seeds were promoted to increase agricultural productivity. While there is still an ongoing debate of the revolution’s impact on hunger alleviation [24,25], some limitations of these interventions can be delineated. The green revolution focused predominantly on the increased production of rice and wheat—staple grains consisting mainly of carbohydrates, modest amounts of protein and a few other nutrients essential to meet human nutritional requirements [26]. Before the revolution, many Asian farmers maintained a more diversified agricultural production system including pulses and legumes. This push to concentrate on a few staple crops may be a contributory factor to the simplified diets, the continued undernutrition in South Asia and widespread hidden hunger [19].

India is a clear example of the failure of these policies to achieve improved food and nutrition security. While it is estimated that between 1966 and 2007, the *per capita* availability of calories increased by nearly 25% in South Asia [27], with India having an amount of food sufficient to feed its population, the prevalence of child malnutrition there is among the highest in the World. While the green revolution improved food productivity significantly, the role of healthcare, childcare, and diverse and quality foods for household food and nutrition security was less emphasized [28]. More detailed analyses have also highlighted that women and children benefited the least from the technological revolution [29,30], and there was insufficient attention towards women’s empowerment and intra-household distribution of resources. However, the revolution had a specific goal—to increase food productivity—and it did accomplish that. One lesson emerging is that massive policy reforms in one sector could be more impactful if other sectors are integrated simultaneously in both policy and practice.

In the beginning of the 1980s, more nutrition elements were incorporated into agricultural policies because of a well defined concept of food security with an emphasis on access [31,32]. Agriculture is an important instrument in reducing hunger in rural areas, by increasing farmers’ income and assets [33]. However, there is plenty of evidence of agricultural projects increasing farmers’ income and assets, which did not succeed in improving child nutrition [34]. Only those programmes which included additional components such as nutrition education, produced improved nutrition outcomes [35].

During the 1990s, agricultural interventions have drawn more attention to nutrition with agricultural production, research, and technology as fundamental for the alleviation of micronutrient deficiencies. This has led to the implementation of “food-based” strategies [35–37]. The new emphasis on nutrition security and public health is well reflected in the definition of food security given at the 1996 World Food Summit (see above). This definition recognizes the presence of a third component of food security, namely food *utilization*, encompassing diet quality and child care practices (Figure 1).

### 3.2. Nutrition Policies

During the 1970s, the focus of nutrition policies and programs was on protein-energy deficiencies. However, as the determinants of poor nutrition become more defined, it was clear that vitamins and minerals play a key role in improved nutrition outcomes. Many pioneers in the nutrition field promoted supplementation and fortification programs to treat obvious deficiencies, such as vitamin A to treat night blindness and iodized salt to treat goitre. These programs are still ongoing and have demonstrated improvements in select micronutrient deficiencies in the developing world.

While there is large empirical evidence of projects based on food supplementation and fortification having improved the nutritional status of the targeted population, this type of intervention (at least in the way it was realized) has some weaknesses. First, these programs have been mainly concerned with the “big three” micronutrients—vitamin A, iron and iodine. Though they are the most widespread as well as those with more significant effects on public health, there are many other essential nutrients whose role in human nutrition has been highly neglected. Second, projects have focused on single nutrient interventions: however, a person is likely to suffer from multiple micronutrient deficiencies [38,39]. What Allen defines as “nutrition isolationism” does not reflect the reality that often people suffer from deficiencies in multiple nutrients [38].

The third limitation is that there is no universal coverage of micronutrient supplements geographically and in populations. Supplying supplements to the most vulnerable and isolated populations is a challenge, as is the case with zinc supplementation in managing diarrhea in sub-Saharan Africa. In population coverage, iron supplements are targeted only to children, adolescents and all women of childbearing age, while vitamin A supplements are targeted only to preschool children and postpartum women [38]. The final limitation of micronutrient supplementation programmes is that they follow a top-down, stop gap approach [36], and take on a short-term solution [17,26]. These technical interventions do address the root causes for the specific micronutrient inadequacies, but do not affect the overall quality of their diet to ensure dietary (along with important non-nutrient elements such as phytochemicals) needs are covered for long lasting health. Therefore, single-nutrient supplements, although critically important in tackling some of the vitamins and minerals deficiencies with large burdens, are unlikely to ensure a sustainable improvement of diets worldwide.

In a few cases supplementation programmes have failed to improve the various nutritional indicators even in the short term. One clear example is the “Accelerated Child Survival and Development programme”, implemented by UNICEF in 11 West African countries between 2001 and 2005. One central pillar of this programme consisted in the delivery of vitamin A supplements. A comprehensive evaluation in Benin and Ghana shows that: (1) there was no reduction in child wasting in Benin due to the intervention, while child wasting increased in Ghana; and (2) there was no significant reduction of childhood mortality due to the project [40].

The above criticisms of supplementation programmes do not imply a rejection of this strategy. Yet, there is a need to move from vertical sectoral interventions to multi-sectoral approaches, where supplementation can be an important component, especially if aimed at addressing multiple micronutrient deficiencies. A comprehensive strategy to address hidden hunger should involve sectors such as agriculture, health, and the environment, with the final goal to improve people’s diets in a sustainable manner. A recent international scientific symposium “Biodiversity and Sustainable Diets,” was an attempt to provide a comprehensive definition of “sustainable diet” and to identify guidelines for programmes aiming at improving diets and micronutrient deficiencies for all [41].

## 4. A Call for Holistic Approaches

Public policies aimed at improving the standards of living of the population are often inadequate for economic growth “corrected” with some social or pro-poor policies. One reason for this is because socio-economic problems such as poverty, lack of education and social services need to be addressed simultaneously to improve nutrition and health in the long-term. Agriculture, health, nutrition, and environment sectors, all examined with a gender lens, need to be viewed as a *unicum* [21,26,42].

In recent years, programs have made an initial effort to move towards more integrated and multi-sectoral solutions to food security, through “food-based” strategies [35,43,44]. In the majority of cases, these interventions have combined a few components, focusing on agricultural production including land usage and ecosystem services with nutrition education, food supplementation, social marketing, or basic primary health care. Some of these programs advocate for the use and consumption of locally available, nutrient-rich foods as a contributor to diverse diets and improved nutritional outcomes [39,45,46].

Lack of diversity is a critical issue particularly in the developing world in which the diets consist mainly of starchy staples, with less access to nutrient-rich sources of food such as animal proteins, fruits and vegetables [37]. Evidence is clear that increasing the consumption of a variety of foods across and within food groups, and within varieties is recommended to ensure adequate intake of essential nutrients and important non-nutrient factors [45]. The biodiversity of foods includes plant based species such as leafy vegetables but also non-timber forest products and other gathered foods, livestock, fish and wetlands species, wild game, and insects. Studies have also shown that dietary diversity is associated with child nutritional status and growth and with socioeconomic status, and links between socioeconomic factors and nutrition outcomes are well known [14,47–49].

Food fortification programmes are often referred to as food-based interventions in improving hidden hunger [43]; however some consider fortification more of a “post processing” intervention that does not necessarily integrate agriculture practices with nutrition outcomes. Fortification consists of adding vitamins and minerals (called “fortifiers”) to common foods (called “vehicle foods”) such as wheat flour, maize flour, rice, sugar, and oil. For this intervention to be effective, it is important to choose the food vehicle carefully to ensure that the vehicle is consumed locally by many. Fortification can be part of a food-based strategy but must be complemented with education to increase the demand of fortified foods in conjunction with other interventions targeting increased diet diversity and quality.

A related strategy but one that falls squarely within agriculture, is biofortification. It refers to the use of traditional crop breeding practices or modern biotechnology to increase the micronutrient concentration in crops. This type of intervention aims at improving the specific micronutrient deficiencies of a target population [17,21,50]. Early studies have demonstrated some success. In Mozambique, production, access and consumption of biofortified orange fleshed sweet potatoes (OFSP) was promoted to tackle vitamin A deficiency in young children [35]. Farmers’ access to improved orange-fleshed sweet potato vines and roots increased, nutrition knowledge improved, demand for this product increased, and a stable market for orange-fleshed potato was promoted [37]. Vitamin A intake increased largely due to increased OFSP intake and serum retinol concentrations improved in young children in the rural communities [51].

Recent work has demonstrated that there is a range of variation in the provitamin A carotenoids (pVACs) contents of bananas, *Musa* fruit (*Musa* cultivars grown and consumed locally, including e.g., the plantains of West Africa and Latin America, the East African highland bananas in East Africa and the Silk bananas in India), with levels approaching those found in the best-performing sweet potatoes and carrot varieties [52]. Next steps will be to demonstrate their effectiveness in decreasing vitamin A deficiency in populations that consume these fruits.

In many food-based programmes, home gardens play an important role in improving access to micronutrient-rich foods. The project “Integrating Homestead Gardening and Primary Health Care Activities in South Africa” is an interesting case study of agriculture, health, and nutrition sector coordination with a goal of improving the nutritional status of the population. The agriculture intervention was only indirectly related to food production since it consisted of vegetable production training [53]. The agriculture component was complemented with an education intervention on the importance of vitamin A in human health, the sources of vitamin A rich foods, and promotion of local production and consumption. The impact included improved nutrition knowledge of mothers, increased consumption of vitamin A rich foods (in particular, carrots, pumpkin, and spinach) amongst children, as well as increased serum retinol concentrations [37].

Another important case study is the homestead food production program implemented by Helen Keller International in four countries characterized by high levels of hidden hunger—Bangladesh, Cambodia, Nepal, and Philippines. This program, which integrates home gardening with animal husbandry, has led to a substantial increase in dietary diversification, in consumption of animal food, and has reduced the prevalence of childhood anaemia [54].

When thinking of food based approaches, animal source proteins are critically relevant. The Nutrition Improvement Project in Vietnam was a community-based project, with two main interventions [43]. The first established a system of productive activities in household gardens consisting of horticulture, pond culture of fish, and small-animal husbandry, while the second activity aimed at increasing mothers’ knowledge of nutrition-related information and practices [55]. The project had success with an increase in production and intake of several micronutrient-rich foods, an improvement of women’s nutrition knowledge and children’s nutritional and health status [55,56].

Traditionally and most commonly, food approaches to addressing food security have been limited in scope. The first limitation is the assumption that production of more diversified foods will lead to higher intake of micronutrients and consumption of more types of food. A local food culture abundant in sources of micronutrient rich foods is essential in these production systems. However, there are many other factors influencing the access to micronutrient-rich foods and people’s dietary choices beyond simply “*staple production followed by consumption*.”

The second limitation is that these strategies have been designed to address the problems of hidden hunger and food insecurity predominantly in rural areas. They are less suitable for urban areas, though the number of urban people that depend on urban agriculture for their food needs is growing rapidly.

The third limitation is that these approaches focus on only two, though crucial sectors—agriculture and nutrition—while leaving aside other important ones. Socio-economic factors such as people’s education, women’s empowerment, cultural beliefs, infant and young child feeding practices, intra-household food distribution and social norms are key determinants of people’s access to adequate and nutritious food, and their dietary behaviours, and can themselves be affected by changes in diets and nutritional status.

Food-based solutions to improve food security have surely contributed to our knowledge on the effectiveness of integrated approaches as these case studies demonstrate, and it is important to emphasize these types of approaches when designing interventions [43,45,57]. These studies provide strong evidence that education and knowledge enhancement are key components as well as working with women as agents of change. The strategic linkages between health, culture, environment and agriculture and nutrition to combat hidden hunger have been described as “food systems”, “field fortification” and “food based” strategies [58,59]. It is in these food systems that ecological concepts can be of particular use to study and optimize the sustainability and nutritional value of the underlying food systems.

## 5. Food and Nutrition Systems

Community and household food security depends on underlying social, economic and institutional factors, which ultimately affect the quantity, quality and affordability of food as well as nutrition, health and wellbeing [60,61]. Conceptually, this can be thought of as a system—a set of elements that function together as collective units which have properties greater than the sum of their component parts. Food systems are dynamic and depend on agriculture, food, eating and health. Policy makers, development agencies and governments often talk about a “health system” or a “farming system” as something that is the total sum of all the organizations, institutions and resources whose primary purpose is to improve health or food production.

A *resilient food and nutrition system* involves people, as consumers, as the central focus. This system also ensures that environmental integrity, economic self-reliance and social well being are maintained and emphasized. A healthy food and nutrition system is self-reliant, controlled, accessible, safe, sustainable, resilient and food-secure. It ensures that links are made between sustainable natural resource management, food production, food consumption and nutritional health [62].

**Figure 2.**
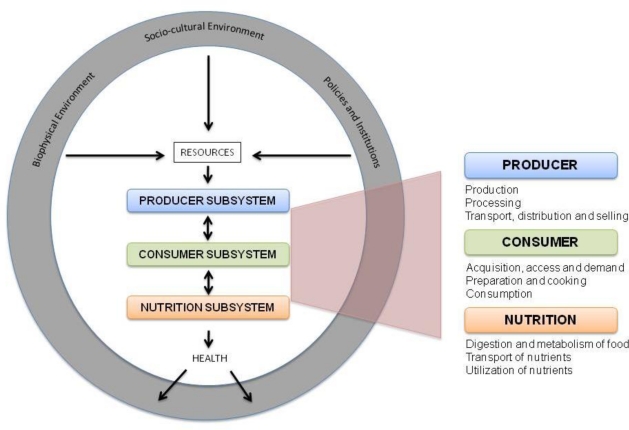
An integrated food and nutrition system (adapted from [62]).

Sobal and colleagues provide a cohesive “food and nutrition system approach”, which can help in the design of more comprehensive policies and programmes [62] which encompasses characteristics from food chain, cycle and web models (Figure 2). Following their definition, this system consists in “the set of operations and processes involved in transforming raw materials into foods and transforming nutrients into health outcomes, all of which functions as a system within biophysical and sociocultural contexts” [62].

The food and nutrition system is characterized by three sub-systems: producer, consumer, and nutrition. This allows for a visualization of a potential sequential order of activities, beginning with food production and ending with food transportation and utilization, with a health outcome. However, the sequential order is not always satisfied, and many relationships between and within the subsystems can follow alternative paths. Moreover, many feedback effects take place: for example, a well-nourished person living in rural areas can engage better in agricultural activity, thus contributing to produce food (with higher productivity).

This system operates with the utilization of resources incorporated into the system in a holistic manner. Resources include biophysical (climate, energy, soil, water, and biodiversity) and sociocultural (economic factors such as markets and capital, cultural values and traditions, individual satisfaction, knowledge and skills, and policy) with *health* as the major outcome of the entire system. Although a system should not be thought of as linear, one starting point could be the use of resources from the biophysical environment, such as agricultural biodiversity, for production of food. These resources are critical for a well nourished population to engage in farming, cooking and digestion of nutrients and foods.

The *producer subsystem* is based on farmers and those engaged in food gathering, herding, fishing, processing, distribution and marketing, products derived from food commodities as well as types of food processor and distribution services. Within the producer subsystem, ensuring nutritious food is available in markets (and marketing of products) is a critical element for ensuring that not only is food available, but that healthier food plays a prominent role from the production side. Though raising smallholder agricultural productivity increases incomes and allows families to purchase more nutritious food such as fruits, vegetables, and animal products, farmer-focused initiatives focusing on the production side can also improve diet diversity and the associated micronutrients lacking in the diet of sub-Saharan populations [63,64]. Diets can be improved through production by breeding of more nutritious crops including traditional local foods and leafy greens and improved nutritionally rich processed foods.

The *consumer subsystem* is made up of consumers and food preparers, products, foods, dishes and meals. Within the consumer subsystem, access to these foods (either grown or purchased), safe and nutritious preparation of foods, and consumption (with equal distribution across a household) are all critical elements of improved nutrition. Typically, poor households subsist on monotonous staple-based diets. They often lack access to micronutrient-rich foods, such as fruits, vegetables, animal source foods (fish, meat, eggs, and dairy products) for consumption. Traditional local foods of high nutrient content and wild food are often underutilized and neglected.

Lastly, the *nutrition subsystem* is made up of food components including macro and micronutrients, fiber, water, and non-nutrient factors. The status of the nutrition subsystem is reliant on the health state of an individual, which is determined by the proper digestion of food, along with the absorption and utilization of nutrients within the body. To have food available and accessible to eat, whether purchased at the market or grown at home, is not the sole solution to food security. A person’s body must be in good physical condition in order to properly *utilize* the food and its micronutrients efficiently for optimum health. Utilization requires not only an adequate diet, but also a healthy physical environment, including safe drinking water, better knowledge of nutritional needs for women and children, improved infant feeding practices, fair intra-household distribution of food, adequate sanitation and hygiene, decreased burden of infectious disease, and the knowledge and understanding of proper care for oneself, for food preparation and safety [44].

A functional food and nutrition system should interact with other systems, such as the health systems, water and sanitation systems, and agriculture production systems, to be effective in reducing hidden hunger [62]. The main problem is to understand how the food and nutrition systems can work at the country policy level and how systems approaches can be implemented on the ground.

Both nutrition and hunger fall within a broader mandate that necessitates the inclusion of agriculture, health, education, water and sanitation, and other departments. This poses clear challenges to leadership and coordination in many developing countries. Too often, no single entity takes primary responsibility for working at the nexus of systems in research, policy and program development. Many governments under invest in programs and efforts to reduce food insecurity, and fail to provide the essential domestic public goods and investments to integrate agriculture and health systems for sustained growth. One important explanation is to be found in the complexity that policy makers and governments encounter in the implementation of multi-sectoral interventions, here deemed as indispensable for tackling the multi-dimensions of food insecurity. There needs to be new political incentives and institutional arrangements, particularly in sectors that are traditionally thought of as very distinct—agriculture and health. It is much easier to design an intervention that looks separately at three core sectors like agriculture, health and nutrition, and does not tackle complex problems that involve land, gender, trade and markets, among others.

Countries should identify context-dependent solutions, which integrate technical prevention and treatment-based interventions with wider efforts to enhance food security and diet diversity. It will be critical to ensure that food and nutrition system approaches are embedded centrally in poverty reduction or development plans or as stand-alone initiatives linked to the overall development vision for countries moving forward. There is plenty of evidence to suggest that in the absence of clear policy, rapid gains are much more limited. But financial support is a must. In countries where resources are scarce, international development assistance will remain an important, temporary solution. Strong evidence and robust tools are available to leverage agriculture that ensures positive nutritional outcomes. Knowledge and expertise to address food security and reduce poverty by leveraging existing food systems are available as well. It is now time to utilize these tools, evidence and knowledge, and to support governments to invest in systems in order to address these issues at global scale.

Communities also play a major role. Community and household food and nutrition security depends on underlying social, economic and institutional factors, which ultimately affect the quantity, quality and affordability of food as well as nutrition, health and wellbeing. By giving communities the tools and knowledge to make choices, a resilient food and nutrition system can be developed that ensures environmental integrity, economic self-reliance and social wellbeing.

## 6. Conclusions

A new focus must be undertaken that addresses the longer-term determinants of nutrition insecurity and hidden hunger. There must be an emphasis on interventions that address food and nutrition security as part of a wider multi-sector strategy tailored to diverse conditions of major agroecological, socioeconomic and epidemiological situations. While underlying determinants of undernutrition have been well understood for decades, the design, testing and scaling of more holistic multi-sectoral packages that combine child care and disease control interventions with food system and livelihood-based approaches has been limited in their development and implementation.
